# Lightweight Graph Neural Network-Driven Acoustic Anomaly Detection Method for Gas Pipeline Leakage Levels in Underground Utility Tunnels

**DOI:** 10.3390/s26134114

**Published:** 2026-06-29

**Authors:** Wei Sun, Yang Li, Jinghu Yang, Ye Cheng

**Affiliations:** 1 China Coal Technology and Engineering Group, Chongqing Research Institute, Chongqing 400037, China; 2 Branch Institute of Emergency Science, Chinese Institute of Coal Science, Beijing 100013, China; yangjinghu@mail.ccri.ccteg.cn (J.Y.); chengye@mail.ccri.ccteg.cn (Y.C.); 3 State Key Laboratory of Disaster Prevention and Ecology Protection in Open-Pit Coal Mines, Chinese Institute of Coal Science, Beijing 100013, China

**Keywords:** underground utility tunnel, gas pipeline, anomaly detection, spatial–temporal graph network

## Abstract

Gas pipeline leakages in urban underground utility tunnels pose a severe threat to public safety. Leakages of varying aperture sizes trigger differentiated risks of diffusion and explosion; thus, achieving precise identification of leakage hole size has become a critical issue in safety management. To address the difficulty of traditional methods in effectively separating the acoustic features of different leakage levels within complex utility tunnel environments, this paper proposes a gas pipeline leakage risk level identification method based on a lightweight Spatial–Temporal Graph Neural Network (ST-GNN). First, relying on a real utility tunnel simulation platform, acoustic signals under different pressures and leakage hole size are collected, and time-frequency magnitude features are constructed through Short-Time Fourier Transform (STFT). Furthermore, each acoustic sample is independently converted into a graph with STFT time frames as nodes, where temporal neighborhood edges and K-nearest neighbor edges jointly encode local dynamics and non-local spectral similarities. This transforms unstructured acoustic signals into graph-structured data that embodies spatial–temporal coupling relationships. Building upon this, a lightweight Chebyshev graph convolutional network is designed to progressively extract discriminative features strongly correlated with leakage levels using multi-layer convolution. Experimental results on the actual utility tunnel simulation platform dataset demonstrate that the proposed method achieves excellent performance in a three-level leakage classification task. The t-SNE visualization reveals the effective separation of features, progressing from complete mixing in the input layer to distinct separation in the output layer. Through multiple training statistics and ablation experiments, the impact of dataset size and the number of network layers on the identification performance is analyzed, validating the robustness of the proposed model under limited samples and the effectiveness of its lightweight structure. This provides a feasible solution for the automated and refined identification of gas pipeline leakage levels in underground utility tunnels.

## 1. Introduction

Urban underground utility tunnels lay electricity, communication, water supply, and thermal pipelines in a centralized manner, making them an important component of modern urban infrastructure. Among them, gas pipelines, as key components of urban operations, occasionally experience damage and leakage due to mechanical stress, external impact, corrosion and aging, and the complex working environment of the underground space. Leak holes of different sizes will lead to varying degrees of gas diffusion and explosion risks: during small-hole leakage, gas continuously escapes at a lower rate, which is not easily detected immediately, and after long-term accumulation, encountering an ignition source may trigger a flash explosion or fire; during medium-hole leakage, the gas injection flow rate increases significantly, easily forming a directional jet; large-hole leakage causes a large amount of gas to be released rapidly in a short period of time, highly likely to form a large-area explosive gas cloud, posing a serious threat to the tunnel structure and ground public safety. Therefore, achieving precise identification of the leak hole size can provide operation and maintenance personnel with a basis for differentiated emergency response and disposal decision-making, which is an important issue urgently needing to be solved in the field of gas pipeline safety management in urban underground utility tunnels [1,2].

Passive detection technology based on acoustic signals, due to its high sensitivity and ability to achieve non-intrusive installation, is particularly suitable for continuous online monitoring in underground utility tunnels. However, the complex acoustic characteristics in the utility tunnel, such as strong reverberation and multi-source noise interference, pose severe challenges to the accurate identification of leakage signals. To address this challenge, researchers have introduced signal processing techniques into the field of pipeline leakage detection, promoting its gradual transition from manual inspection to automated analysis. Short-Time Fourier Transform (STFT), as a basic signal processing method, is used to convert time-domain leakage signals into time-frequency domain representations. For example, Lay-Ekuakille et al. conducted spectral analysis of acoustic leakage signals in urban water supply networks using STFT, verifying the effectiveness of this method in pipeline leakage detection [3]. In addition to STFT, other time-frequency analysis tools such as wavelet transform (WT) and Hilbert-Huang transform (HHT) have also been successfully applied to pipeline leakage detection, offering multi-resolution capabilities and adaptive basis functions that are particularly effective for non-stationary leakage signals [4,5]. Aiming at the limitation of the fixed STFT window, Empirical Wavelet Transform (EWT) achieves finer frequency band extraction by adaptively dividing the signal spectrum [6]. On this basis, Xiao Qiyang et al. introduced EWT into the task of pipeline leakage acoustic signal analysis, and by extracting leakage-sensitive components after EWT decomposition of leakage acoustic/vibration signals, achieved good localization accuracy and minor leakage detection performance [7,8]. At the same time, Empirical Mode Decomposition (EMD) and its improved methods (such as EEMD, CEEMDAN) have shown unique advantages in non-stationary signal processing, and the pipeline leakage detection denoising algorithm based on EEMD-PRT effectively improves the stability and reliability of IMF components by introducing phase randomization technology [9,10]. Although the aforementioned signal processing methods significantly improve the automation level of leakage detection, the features they extract are mostly statistics geared towards binary detection (leakage/normal), and their representation capabilities are obviously insufficient for distinguishing the subtle acoustic differences corresponding to different leakage hole size. The final risk level determination still needs to rely on manual experience or rules based on fixed thresholds, making it difficult to achieve automated and refined grading of leakage hole size.

In recent years, with the rise of artificial intelligence technologies such as deep learning, pipeline leakage detection has begun to deeply develop towards intelligence and automation. Convolutional Neural Networks (CNNs), Recurrent Neural Networks (RNNs), and their variant models can autonomously extract leakage features directly from input data for classification. In CNN applications, Wang Xiufang et al. proposed a pipeline leakage aperture identification method based on dynamic depthwise separable convolutional neural networks, achieving stronger feature expression capabilities through dynamic convolutional layers and channel attention mechanisms [11]; the leakage identification model proposed by Luo Zhengshan et al., which combines EEMD with an Improved Convolutional Neural Network (ICNN), achieved an average recognition accuracy of 98.25% under multiple working conditions [12]. In terms of acoustic-image fusion, a multi-condition pipeline leakage diagnosis method based on a whale optimization evolutionary convolutional neural network achieved accurate leakage state differentiation under five working conditions, with diagnostic accuracy significantly higher than traditional CNN models [13]. In RNN and LSTM applications, Mishra et al. proposed a pipeline leakage detection technology based on acoustic emission feature sequences and LSTM networks, capturing the temporal dynamic patterns triggered by leakage by constructing temporally modulated short-time descriptors, and achieved high classification accuracy on experimental signals [14]; a non-metallic pipeline leakage level identification strategy based on PCA-Bi-LSTM reached an accuracy of 98.6% in the leakage level identification task, significantly higher than that of a unidirectional LSTM network [15]. These deep learning methods have laid a solid foundation for the automated detection of pipeline leakage. In recent years, Transformer-based models [16], generative adversarial networks (GANs) [17], and self-supervised learning frameworks have also been introduced into pipeline leakage detection [18], showing promising potential in handling long-range dependencies, imbalanced data, and limited-label scenarios.

However, existing deep learning-based pipeline leakage detection methods still have two key limitations. On the one hand, predicting continuous acoustic signals merely as time-series samples will lead to the loss of spatial–temporal correlations inherent in the acoustic differences corresponding to different leakage hole size. On the other hand, when converting data into two-dimensional images to fit convolutional neural networks, although time-frequency spatial features can be extracted, restricting the data to grid-like structural features may lose the non-Euclidean topological relationships in the original signal. These limitations cause the leakage features under different pressure conditions to easily confuse with each other in weak feature working conditions, thereby affecting the accuracy of level identification and the effect of field applications [19]. In fact, many real-world signals, including pipeline acoustic emissions, exhibit inherent graph-like or non-Euclidean structures that cannot be adequately represented by regular grids or sequences [20]. Graph neural networks have been increasingly adopted in industrial anomaly detection [21], further motivating their use for pipeline leakage modeling.

As a graph neural network structure developed in recent years, Spatial–Temporal Graph Neural Network (ST-GNN) can effectively process non-Euclidean spatial data and exhibits unique advantages in the field of pipeline leakage detection. Şahin and Yüce employed a Graph Convolutional Network (GCN) for leakage and blockage detection in water supply pipeline networks, achieving a 91% fault detection accuracy across five different scenarios [22]. Zhang et al. proposed a natural gas pipeline leakage detection and localization method based on a deep probabilistic graph neural network. By modeling sensor spatial dependencies through an attention-based graph neural network and combining variational Bayesian inference, it achieved leakage localization without anomaly labeled data, with an AUC reaching 0.9484 [23]. Regarding leakage detection in water distribution networks, Zhang et al. proposed an Algorithm-Informed Graph Neural Network (AIGNN). By simulating the Ford–Fulkerson maximum flow algorithm for pre-training, the model extracted more generalized task-related features, achieving superior generalization capabilities compared to conventional GNNs in leakage detection and localization tasks [24]. Lu et al. proposed an end-to-end graph neural network framework based on intelligent graphs, converting acoustic emission signals into graph representations, and achieved a localization accuracy of over 93% under 2–5 MPa pressure conditions [25]. Moreover, graph attention networks (GATs) and dynamic spatial–temporal graph networks have also been successfully applied to leak detection in water distribution networks and real-time condition monitoring of industrial pipelines [26,27], confirming the general advantage of GNNs in modeling spatiotemporal dependencies. These studies demonstrate the immense potential of graph neural networks in spatial–temporal dependency modeling for pipeline systems. However, it is worth noting that current applications of graph neural networks in the pipeline leakage domain are mainly concentrated on binary detection or leakage localization tasks. There is no research yet applying them to the fine-grained identification of pipeline leakage levels (such as the three-level classification of small hole, medium hole, and large hole), especially in the special scenario of an underground utility tunnel which is closed, highly reverberant, and subject to multi-source interference; research in this direction remains a blank.

With the development of edge computing and the Internet of Things, lightweight deep learning models have become a key direction for industrial field deployment due to their low latency and low power consumption [28,29]. Addressing the aforementioned research gaps, this paper applies ST-GNN to the task of gas pipeline leakage risk level identification in urban underground utility tunnels, aiming to solve the problem that the features of different levels of leak holes are difficult to separate effectively under complex working conditions. The main contributions of this paper are as follows:

(1) For the first time, a spatial–temporal graph construction method is applied to the processing of gas pipeline acoustic signals in underground utility tunnels. It converts the experimentally collected acoustic signals into graph-structured data and simultaneously encodes the spatial–temporal topological relationships between samples and the time-frequency features of the acoustic signals within the graph structure, filling the research gap of using graph neural networks for pipeline leakage level identification.
(2) A lightweight spatial–temporal graph convolution feature extraction structure is proposed to realize the joint modeling of spatial–temporal coupling relationships, and this lightweight structure is advantageous for subsequent embedded deployment.
(3) The application effectiveness of this model in multi-classification scenarios is verified on an actual simulation platform dataset. The impacts of the number of layers and the number of training samples on detection performance are analyzed, providing a basis for engineering deployment.

The structure of this paper is organized as follows: Section 2 introduces the collection method of pipeline gas leakage acoustic signals, the grading standards, and the traditional time-frequency features of leakage signals at different levels. Section 3 presents the specific theoretical basis, architecture, and implementation of the spatial–temporal graph network model. Section 4 demonstrates and analyzes the numerical experimental results of the lightweight spatial–temporal graph neural network. Section 5 summarizes the entire paper and provides an outlook on future work.

## 2. Acoustic Signals of Gas Pipeline Leakage in Utility Tunnel Environments

### 2.1. Construction of Gas Pipeline Leakage Acoustic Signals in Simulated Utility Tunnel Environments

Obtaining a high-quality dataset of pipeline leakage acoustic signals is a necessary prerequisite for research into leakage detection and level identification. In recent years, the academic community has formed two mainstream technical routes for acquiring these signals: real-world leakage data collection and numerical simulation.

In terms of real leakage data collection, the GPLA-12 dataset released by Li and Yao is one of the larger public datasets in the field of pipeline leakage acoustics, covering 684 training/testing acoustic signals and 12 classification categories; this dataset has been widely used in research on gas pipeline leakage detection based on acoustic emission signals. Li Yuxing et al. designed a high-pressure gas transmission pipeline leakage test device based on the transient model method and the acoustic wave leakage detection method, verifying the generalizability of model tests through similarity analysis [30]. Liu et al. designed a complete high-pressure gas pipeline leakage experimental device and acoustic data acquisition system based on the acoustic wave leakage detection method, using CFD simulation to guide the selection of experimental parameters [31]. While these experiments provided a large amount of valuable measured data, most were conducted on relatively ideal open pipeline structures with varying sensor deployment methods, failing to fully simulate real engineering scenarios such as multi-pipeline parallelism and restricted spatial reverberation in underground utility tunnels.

In terms of numerical simulation, the combination of Computational Fluid Dynamics (CFD) and aeroacoustic theory provides another important tool for studying pipeline leakage acoustic signals. By using commercial CFD software for high-precision simulation of the leakage flow field and extracting sound source characteristics through acoustic analogy methods, the limitation of experimental data coverage can be mitigated, allowing for the flexible simulation of various combinations of leakage hole size, internal pressures, and medium conditions. Regarding leakage aeroacoustic source modeling, Liu et al. studied the generation mechanism of aeroacoustics during leakage and established a simulation model in CFD software to obtain flow field and sound field data [32]. Recent studies have further employed Large Eddy Simulation (LES) to calculate the non-steady flow field during gas pipeline leakage and combined it with the Möhring acoustic analogy method to extract aeroacoustic sources, discovering that the leakage source exhibits quadrupole characteristics [33]. Li et al. utilized CFD methods to study the acoustic wave propagation model of pipeline leakage, constructing an amplitude attenuation model and a waveform diffusion model, which provided a theoretical basis for acoustic wave leakage localization methods [34]. Ayyildiz et al. used CFD simulation to analyze micro-hole leakages of 1.27–3.3 mm, studying leakage flow field characteristics under low-pressure conditions, including flow leakage, pressure distribution, and velocity profiles, and used Power Spectral Density (PSD) and Fast Fourier Transform (FFT) to predict sound pressure changes and acoustic oscillations and turbulence behavior around the leak hole [35].

Despite the unique advantages of numerical simulation in terms of operational flexibility, it faces fundamental challenges. The simplified assumptions of CFD simulation regarding actual physical processes introduce systemic biases, and the authenticity and reliability of its calculation results always require calibration and validation with experimental data [36]. More critically, multi-path interference of sound propagation paths and strong reverberation effects in utility tunnels are often oversimplified or completely ignored in existing simulation models, making it difficult for simulated data to truly reflect sound propagation characteristics in the semi-enclosed space of an underground utility tunnel [37]. Furthermore, CFD simulations typically require significant computational resources, especially when using high-precision turbulence models like LES, making the computational cost extremely expensive and difficult to promote on a large scale in engineering practice [38].

**Figure 1:**
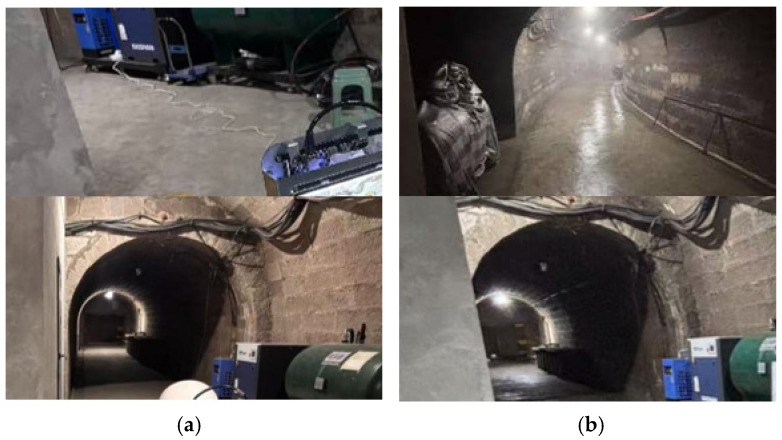
Seasonal environmental differences in the utility tunnel (**a**): Winter; (**b**): Summer.

The aforementioned review indicates that while existing data acquisition methods have specific application scenarios, none adequately adapt to the actual requirements of gas pipeline leakage detection in urban underground utility tunnels. Pipeline leakage is a low-probability event; operational data collection struggles to cover complex environments and multi-condition factors, while numerical simulation fails to accurately simulate the sound propagation characteristics of enclosed tunnel spaces, particularly humidity differences caused by seasonal changes. To solve these problems, this study built a gas pipeline leakage acoustic signal simulation platform based on a real utility tunnel space in Southern China. As shown in Figure 1, the city where this utility tunnel is located is in a subtropical humid monsoon climate zone with large temperature differences across four seasons—rainy summers and relatively dry winters—covering many environmental impact factors. Compressed air was used as the leakage gas source instead of natural gas to ensure experimental safety.

**Figure 2:**
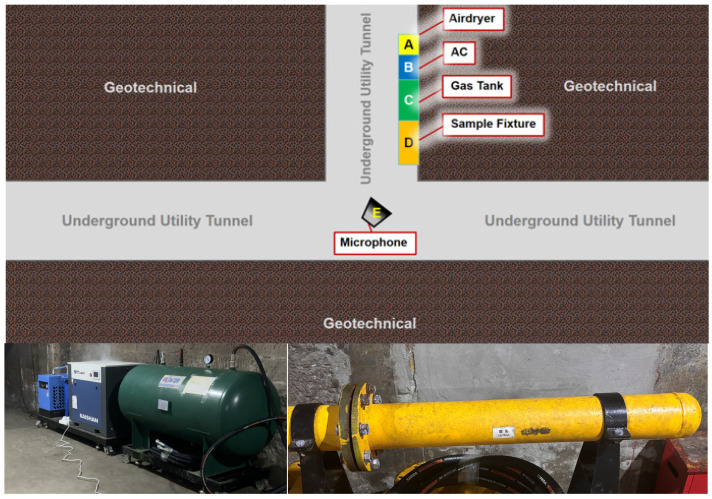
Physical image of the simulated experimental platform and its installation position in the utility tunnel.

The experimental platform mainly consists of four parts, as shown in Figure 2: (A) An air dryer, used to handle the impact of the high-humidity tunnel environment on the air compressor; (B) An air compressor, providing a high-pressure gas source for the experiment; (C) A large buffer gas tank, providing a stable pressure source when the compressor is off to avoid interference from compressor noise during acquisition; (D) Pipeline samples with man-made defects, used to simulate the damaged state during pipeline leakage.

**Figure 3:**
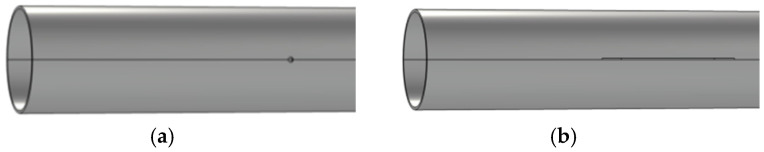
3D structural diagrams of designed pipeline defects (**a**): hole defect; (**b**): slit defect.

As shown in Figure 3, the experiment employed two leak types: one is a circular-like breakage, simulating defects caused by conventional corrosion; the other is a slit-like notch, often appearing under conditions of stress corrosion or mechanical collision. To ensure sample diversity and representativeness, multiple specifications of defect sizes were designed, as shown in Table 1.

### 2.2. Classification of Pipeline Leakage Levels

The reasonable classification of pipeline leakage levels serves as the foundation for the refined identification of leakage level. Currently, academia and the engineering community primarily classify gas pipeline leakage levels based on two methods: leakage aperture size or the proportion of leaked gas volume.

Classification based on leakage aperture directly determines leakage flow rate, jet velocity, and sound source intensity, and is closely related to the severity of the leakage consequences. Taking high-pressure urban gas pipelines as an example, leakage modes are typically divided into four levels: small hole leakage (5 mm), medium hole leakage (25 mm), large hole leakage (100 mm), and pipeline rupture (300 mm). Additionally, finer-grained classification methods, such as pinhole leakage (1–3 mm) and micro-hole leakage (3–10 mm), are used for identifying minor leakages in equipment, pipeline flanges, and instrument joints. Classification based on the volume of leaked gas often uses the ratio of leaked gas to the total transmission volume as the evaluation criterion: small leaks involve a volume less than 3% of the total transmission, medium leaks range from 3% to 10%, and large leaks exceed 10%.

Considering the research objectives and practical engineering requirements, this paper adopts an aperture-based classification method. Accounting for the diversity of leak hole shapes, leakage area is used as the basis for division, classifying gas pipeline leakages into three levels:

Level 1 (Leakage area < 4 mm^2^): Gas escapes continuously. In practical engineering, this usually corresponds to preliminary defects such as local corrosion perforation or micro-cracks. Due to rust spots or uneven pipe surfaces, the actual leak hole is difficult to find visually; however, activating explosion-proof axial fans for strong ventilation can effectively reduce the risk of explosion.

Level 2 (Leakage area ≈ 4 mm^2^ to 8 mm^2^): The gas jet velocity increases significantly, easily forming a directional jet. This typically corresponds to expanded local damage or medium-sized external force injuries. Leakage rates and volumes rise significantly, and forced ventilation or water mist dilution schemes struggle to effectively mitigate explosion risks, posing a direct threat to the structural safety of the utility tunnel.

Level 3 (Leakage area > 8 mm^2^): A large amount of gas is released rapidly in a short time. Even with forced ventilation, the discharged gas easily accumulates on the surface and forms a large-area explosive gas cloud, posing a severe threat to the tunnel structure and ground public safety. This usually corresponds to major accident conditions such as severe pipeline damage or joint failure, requiring the immediate activation of emergency plans and external support.

This three-level classification system fully considers the engineering consequences, providing a physically meaningful classification basis for subsequent acoustic-signal-based leakage level identification, as well as clear and distinguishable supervision labels for model training. In the following experiments, Level 1, Level 2, and Level 3 are, respectively, mapped to Class 0, Class 1, and Class 2 as model labels.

### 2.3. Time-Frequency Characteristics of Acoustic Signals Under Different Leakage Conditions

The acoustic signals generated by pipeline leakage are a generalized acoustic emission phenomenon formed by turbulence shear and wall interaction as gas is ejected from the leak hole at high speed. The time-frequency characteristics of these acoustic signals are closely related to factors such as leakage aperture, internal pipeline pressure, and leak hole type.

**Figure 4:**
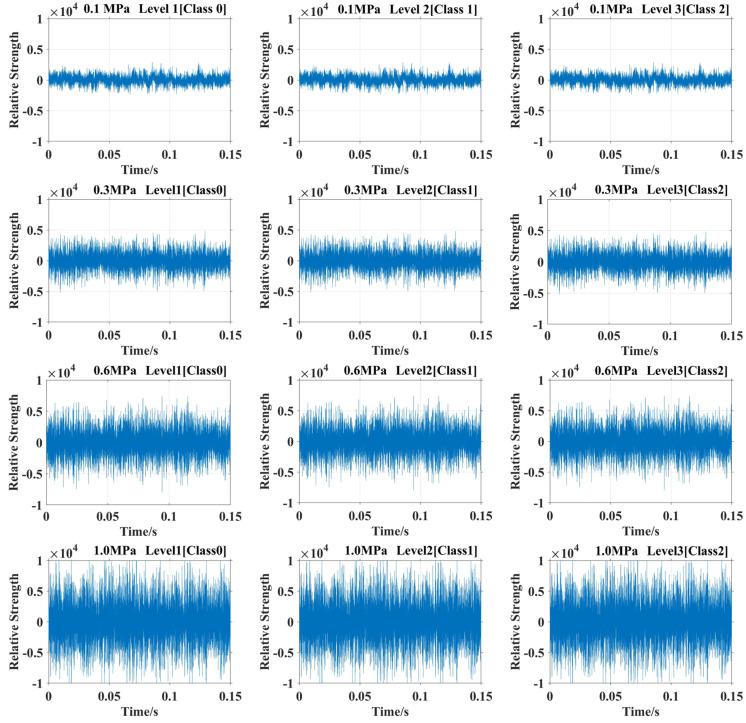
Original acoustic signals under different conditions (From (**left**) to (**right**): different leakage levels; from (**top**) to (**bottom**): increasing pipeline pressure).

Based on the simulated experimental platform described above, this study systematically analyzed the impact of internal pipeline pressure and leakage levels on the time-frequency characteristics of acoustic signals. From a pressure gradient perspective, as shown in Figure 4, the signals exhibit significant nonlinear evolution characteristics in both the time and frequency domains. In the time domain, signals under low-pressure conditions are dominated by stable random noise, with relative strength peaks maintained at a magnitude of $0.2\times 10^{4}$; the waveform oscillations are gentle and the transitions between peaks and valleys are smooth, indicating low vibration excitation energy and a system operating within a linear vibration range. As pressure gradually increases, the peak relative strength grows exponentially, reaching $0.8\times 10^{4}$ under high-pressure conditions; the proportion of sharp peaks and valleys in the waveform increases significantly, and the intensity of oscillations is markedly enhanced.

**Figure 5:**
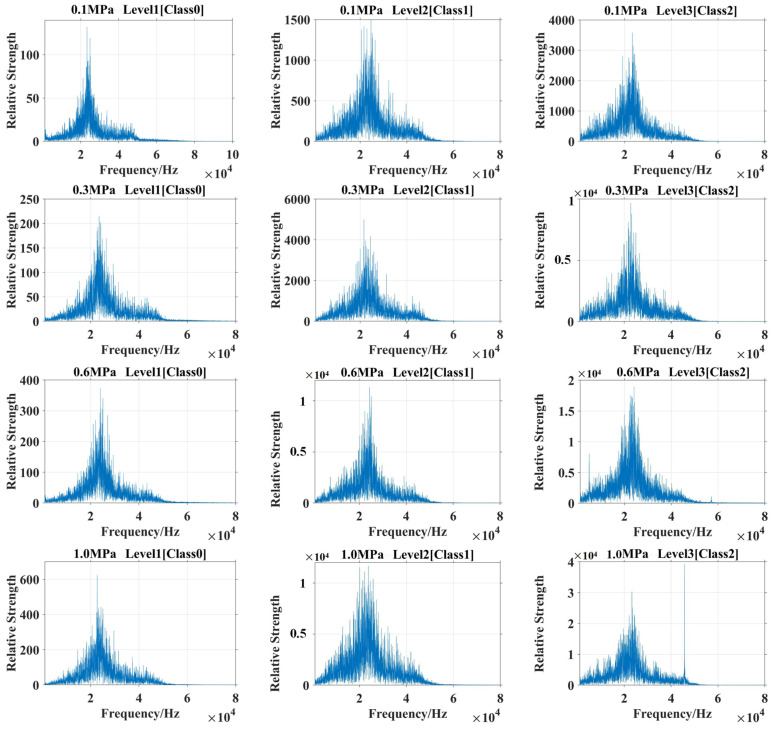
Energy distribution in the frequency domain (From (**left**) to (**right**): different leakage levels; from (**top**) to (**bottom**): increasing pipeline pressure).

As shown in Figure 5, in the frequency domain, the spectral main peak under low-pressure conditions is concentrated in the 20–30 kHz range, with a peak relative strength of approximately 150. The spectral distribution is relatively broad and the energy is dispersed, with a low proportion of high-frequency harmonic components, which is consistent with the spectral characteristics of linear vibration signals. As pressure increases, the intensity of the main spectral peak grows, with relative strength peaks exceeding 12,000 under high-pressure conditions. Simultaneously, the spectral distribution range gradually broadens, and the number and intensity of secondary peaks increase. This indicates that increasing pressure leads to a nonlinear amplification of excitation energy at the vibration source, causing the system vibration mode to transition from linear to nonlinear, increasing the proportion of high-frequency harmonics, making the waveform more intermittent and bursty, and causing spectral energy to diffuse into higher frequency regions.

There is a significant synergistic effect between pressure and the leak hole on the signal. Under coupled conditions of high pressure and large leak holes, the time-domain signal simultaneously exhibits high-intensity amplitudes and strong regular fluctuations; the relative strength peak can reach 8000, and the repetition frequency of periodic pulse clusters increases significantly. In the frequency domain, the main spectral peak intensity reaches its maximum value, while the number and intensity of secondary peaks are significantly enhanced, further broadening the spectral distribution range. This indicates that the joint action of pressure and the leak hole amplifies the nonlinear vibration characteristics of the system, enhancing the coupling effect between random disturbances and periodic excitations, thereby making signal anomaly features even more prominent in both time and frequency domains.

## 3. Lightweight Spatial–Temporal Graph Network Model for Leakage Sound Classification

The time-frequency analysis results presented above indicate that acoustic signals corresponding to different leakage levels exhibit certain distributional differences in both the time and frequency domains. However, these differences manifest complex nonlinear coupling characteristics under varying pressure conditions, making it difficult to achieve refined leakage level identification using only traditional time-frequency features. On one hand, although the energy distributions of small-hole and large-hole leakages differ, their frequency bands overlap, and ambient noise together with tunnel reverberation further obscure feature boundaries. On the other hand, signal features of the same leakage level may drift under different pressure conditions, rendering fixed-threshold classification methods ineffective. Therefore, a modeling approach that can simultaneously capture intra-signal temporal dependencies and spectral structural similarities is required. Graph neural networks, with their powerful representation capability for non-Euclidean data, offer a new perspective for modeling complex relationships among time frames. Motivated by this, we propose a lightweight spatial–temporal graph neural network that independently constructs a graph structure for each acoustic sample and employs Chebyshev graph convolutions to extract deep discriminative features, thereby achieving high-precision leakage level identification.

### 3.1. Graph Convolutional Networks

A graph convolutional network is a class of neural networks specifically designed to process graph-structured data. A graph can be represented as G = (V,E,X,A), where $V\;=\;\{v_{i}|i\;=\;1,2,\ldots ,n\}$ is the set of nodes, E⊆V×V is the set of edges, $X\;\in \;R^{n\times d}$ is the node feature matrix (n: number of nodes, d: feature dimension per node), and $A\in \{0,1{\}}^{n\times n}$ is the adjacency matrix. In the leakage sound classification task of this work, each sample is modeled as an independent graph, where nodes correspond to the time frames of the short-time Fourier transform and node features are frequency-domain magnitude vectors.

Spectral graph convolution leverages the eigen-decomposition of the graph Laplacian matrix to perform convolution operations. Traditional spectral convolution suffers from two limitations: the convolution kernel is global with a number of parameters equal to the number of nodes n, and its computational complexity is as high as $O(n^{2})$. To address these issues, Defferrard et al. [39]. approximated the graph convolution kernel using a K-order Chebyshev polynomial expansion. First, the filter is parameterized as a polynomial (1).

(1) $$g_{\theta }(\Lambda )\approx \sum_{k=0}^{K-1}\theta_{k}\Lambda^{k}$$

Here, k is the highest order of the polynomial, reducing the number of parameters from *n* to K, but the complexity remains $O(n^{2})$. To further reduce computational cost, the Chebyshev polynomial $T_{k}(x)$ is used for approximation (2).

(2) $$g_{\theta }\prime (\Lambda )\approx \sum_{k=0}^{K-1}\theta_{k}T_{k}\left(\tilde{\Lambda }\right)\;$$

Here, $\theta_{k}$ are trainable Chebyshev coefficients, and $T_{k}(x)$ is defined recursively.

(3) $$\left\{\begin{array}{c} T_{0}(x)=1,T_{1}(x)=x\\ T_{k}(x)=2xT_{k-1}(x)-T_{k-2}(x),k\geq 2 \end{array}\right.\;$$

The matrix $\tilde{\Lambda }$ is the normalized diagonal matrix of eigenvalues (4).

(4) $$\tilde{\Lambda }=\frac{2\Lambda }{\lambda_{\max}}-I_{N}$$

Here, $\lambda_{\max}$ the largest eigenvalue of the Laplacian matrix. This scaling ensures that the eigenvalues of $\tilde{\Lambda }$ lie in the interval [−1,1], as required by the Chebyshev polynomial recurrence. In practice, this normalization and the subsequent Chebyshev spectral convolution are efficiently implemented by the ChebConv layer in PyTorch 2.11.0 Geometric, which internally computes the scaled Laplacian and performs the recursive filtering operation. By convolving the Chebyshev polynomial filter with the input feature matrix X, multi−order neighborhood feature aggregation is achieved (5).

(5) $$M=\sum_{k=0}^{K-1}\theta_{k}UT_{k}\left(\tilde{\Lambda }\right)U^{T}X\;$$

Graph convolution can be viewed as filtering the graph signal without changing the feature dimension. To change the feature dimension, a trainable weight matrix can be multiplied after the graph convolution (6).

(6) $$X^{i}=Cheb\left(M,W^{i}\right)=MW^{i}$$

Here, $W^{i}$ is the trainable weight matrix of the i-th layer, and Cheb(⋅,⋅) denotes the Chebyshev convolution function.

Based on the above theoretical foundation, we design a lightweight Chebyshev graph convolutional classifier using the ChebConv implementation from PyTorch Geometric, which handles the Laplacian scaling and Chebyshev polynomial recursion internally. The classifier consists of three Chebyshev graph convolutional layers. The first layer maps the input node features (dimension F=129) to a 64-dimensional hidden space, the second layer further compresses them to 32 dimensions, and the third layer outputs a 16-dimensional node representation. A global mean pooling operation then aggregates all node features into a graph-level vector, which is fed into a linear layer to produce classification scores for the three leakage levels, and finally LogSoftmax is applied to obtain the probability distribution. The entire model can be formalized as (7).

(7) $$\left\{\begin{array}{lll} H_{1} & =ReLU({ChebConv}_{1}(X,E;\Omega_{1})),\;\Omega_{1}\in R^{3\times 129\times 64} & \\ {\tilde{H}}_{1} & =Dropout(H_{1},\;p=0.3) & \\ H_{2} & =ReLU({ChebConv}_{2}({\tilde{H}}_{1},E;\Omega_{2})),\;\Omega_{2}\in R^{3\times 64\times 32} & \\ {\tilde{H}}_{2} & =Dropout(H_{2},\;p=0.3) & \\ H_{3} & ={ChebConv}_{3}({\tilde{H}}_{2},E;\Omega_{3}),\;\Omega_{3}\in R^{3\times 32\times 16} & \\ h & =\frac{1}{T}\sum_{t=1}^{T}H_{3,\;t,\cdot }\in R^{16} & \\ {\tilde{h}}_{G} & =Dropout(h,\;p=0.3) & \\ z & =W{\tilde{h}}_{G}+b,\;W\in R^{3\times 16},\;b\in R^{3} & \\ \hat{y} & =LogSoftmax(z) & \end{array}\right.$$

$H_{l}\in R^{T\times d_{l}}$ denotes the node feature matrix output by the l-th Chebyshev graph convolutional layer, $d_{l}$ is the output feature dimension of that layer. ${\tilde{H}}_{l}$ is the result of applying Dropout. $\Omega_{l}\in R^{3\times d_{l-1}\times d_{l}}$ represents all trainable parameter tensors of the l-th ChebConv layer, which encapsulate both the Chebyshev polynomial coefficients and the feature projection weights. Global mean pooling compresses the node features $H_{3}$ of the third layer along the node dimension into a graph-level representation h in $R^{16}$, which is then also subjected to Dropout to produce ${\tilde{h}}_{G}$. The weight matrix $W\in R^{3\times 16}$ and bias $b\in R^{3}$ of the linear classification layer map the graph-level feature to classification scores z, and finally LogSoftmax yields the log-probability prediction $\hat{y}$ for the three leakage levels.

It should be noted that the effective operation of graph convolutional layers heavily depends on the reasonable construction of the input graph structure. Therefore, the following subsection elaborates on the generation of graph data from raw acoustic signals.

### 3.2. Spatial–Temporal Graph Construction Method for Single-Sample Acoustic Signals

In contrast to most graph neural network approaches that treat all samples as nodes in a global graph, this paper adopts a per-sample independent graph construction strategy. Each leakage acoustic sample is preprocessed to generate an independent graph data structure. This inductive graph construction method is more suitable for online monitoring scenarios and preserves the internal time-frequency structure of each sample. Specifically, the graph construction process begins with time-frequency analysis.

For a given leakage acoustic signal x(t), the short-time Fourier transform is first applied. A Hanning window is used as the window function, with window length set to L=256, overlap length set to 128, and sampling rate set to 80 kHz. The short-time Fourier transform is defined as (8).

(8) $$S(\tau ,f)=\int_{-\infty }^{+\infty }x(t)\cdot \omega (t-\tau )\cdot e^{-j2\pi ft}dt$$

Here, S(τ,f) is the complex time-frequency matrix, f denotes frequency, τ denotes the time frame, and ω(t−τ) is the window function. The Hanning window is expressed as (9).

(9) $$\omega (t)=0.5\left[1-\cos\left(\frac{2\pi t}{L}\right)\right]$$

Here, L is the window length. The transformation yields a complex time-frequency spectrum matrix $S\in C^{T\times F}$, where T is the number of time frames and F=129 is the number of frequency bins. The magnitude spectrum yields a real matrix $X=|S|\in R^{T\times F}$, with each row $x_{t}\in R^{F}$ corresponding to the frequency-domain feature vector of a time frame. In this work, the STFT magnitude spectrum is directly used as the node feature matrix, as experiments show that the magnitude spectrum alone provides sufficient discriminative information for distinguishing different leakage levels, thereby avoiding the extra computational overhead of explicit power spectral density estimation.

Once node features are obtained, the connections between nodes—i.e., the graph edges—need to be defined. Each time frame is treated as a node in the graph, so the total number of nodes is T. Two complementary strategies are employed to define graph edges. The first is temporal neighborhood edges: each node is connected to its two preceding and two succeeding frames, i.e., node $v_{t}$ is connected to $v_{t-2},v_{t-1},v_{t+1},v_{t+2}$. This bidirectional edge structure enables the model to capture short-term dynamic evolution patterns of the leakage acoustic signal along the time axis. The second strategy is $K_{nn}\text{-}nearest$ neighbor edges: to capture non-local similarities between different time frames (e.g., harmonic structures or formant patterns), the cosine similarity between all pairs of node feature vectors is computed. Cosine similarity is defined as $cosine(i,j)=\frac{x_{i}\cdot x_{j}}{\|x_{i}{\|}_{2}\|x_{j}{\|}_{2}}$. Each node is connected to the $K_{nn}$ most similar other nodes, with $K_{nn}=10$. The union of the two types of edges forms the final edge set E, thereby completely defining the graph topology. Ultimately, each sample is transformed into a graph $G_{i}=(X_{i},E_{i})$, where $X_{i}\in R^{T\times F}$ and the adjacency relations are recorded by an edge index matrix. This per-sample independent graph construction strategy avoids the huge computational cost associated with a global graph and is naturally suited for inductive learning in online monitoring scenarios.

### 3.3. Model Training and Evaluation Strategy

After obtaining the graph-structured data and determining the network architecture, the training procedure and performance evaluation methods need to be further specified. The goal of model training is to minimize the discrepancy between predicted and true labels. This paper adopts the negative log-likelihood loss function, defined as (10).

(10) $$L=E_{i\in V_{train}}\left[H\left(y_{i},{\hat{y}}_{i}\right)\right]$$

Here, $V_{train}$ denotes the set of training samples, H(⋅,⋅) is the cross-entropy loss function, $y_{i}$ is the true label, and ${\hat{y}}_{i}$ is the log-probability output by the model. This loss function is equivalent to the cross-entropy loss for classification tasks.

The total sample is collected from the simulated utility tunnel platform (covering three leakage levels). Each sample is independently converted into a graph following the method described in Section 3.2. The Adam optimizer is employed with an initial learning rate of 0.001. A cosine annealing learning rate scheduler is used to gradually reduce the learning rate over a total of 300 training epochs.

Model performance is evaluated on the test set using multiple metrics, including accuracy, macro-averaged $F_{1}$ score, macro-averaged AUC, and Matthews correlation coefficient. The confusion matrix and receiver operating characteristic curves are also computed to provide a detailed analysis of the model’s classification behavior across the three leakage levels. These evaluation methods comprehensively reflect the model’s ability to identify different levels of leakage risk. Based on the model design, graph construction method, and training strategy described above, the next section will comprehensively validate the proposed method through numerical experiments.

**Figure 6:**
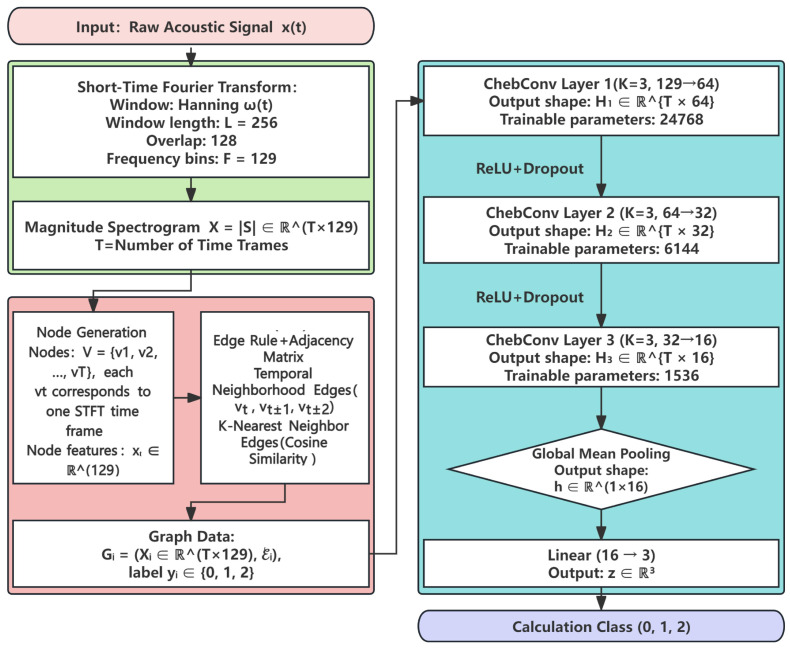
Architecture of the proposed lightweight Chebyshev graph convolutional network.

The constructed neural network model is illustrated in Figure 6, and the next section will comprehensively validate the proposed method through numerical experiments.

## 4. Numerical Results and Analysis

All experiments are conducted on a workstation equipped with an Intel Core i7-12700H processor (14 cores, 20 threads) and 32 GB of DDR4 memory operating at 4800 MHz, without GPU acceleration. The proposed lightweight graph neural network comprises 32,611 trainable parameters. The dataset consists of 960 acoustic samples, randomly partitioned into training, validation, and test sets at a ratio of 40%:30%:30%. Unless otherwise specified, all experiments are performed under this unified configuration. The dataset can be found in the Supplementary Materials of this article.

### 4.1. Clustering Effect Analysis

**Figure 7:**
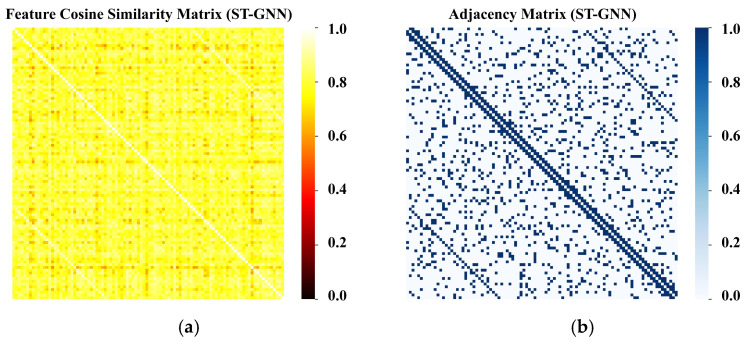
Learned graph properties after training: (**a**) node feature similarity heatmap; (**b**) graph edge connection matrix heatmap.

After the training of the graph neural network is completed, the calculation results are shown in Figure 7. The node feature similarity heatmap exhibits a large bright yellow area, indicating that most node pairs have cosine similarities between 0.75 and 0.95. This suggests that after graph neural network transformation, node embeddings become globally convergent and highly clustered. Despite the sparse graph topology (with only a few edges per node), the message passing mechanism effectively propagates information, enabling most nodes to learn similar global features. A few small orange-red blocks (similarities 0.4–0.7) indicate the presence of nodes with stronger discriminative power, while the absence of large dark regions confirms no severe feature polarization. The edge connection matrix heatmap shows a dark blue diagonal band (strong self-connections) and scattered off-diagonal blue blocks, corresponding to non-local similarities captured by K-nearest neighbor edges. This demonstrates that the learned graph structure preserves both node-specific information and meaningful inter-frame similarities, facilitating effective feature extraction for leakage level identification.

**Figure 8:**
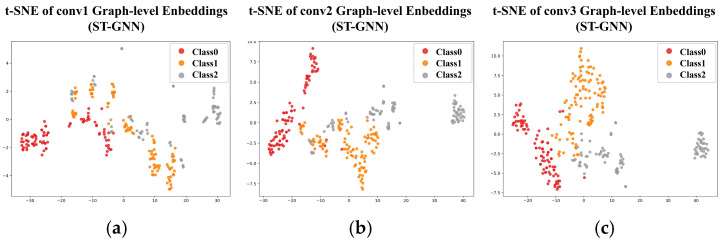
t-SNE visualization of graph-level embeddings at each layer: (**a**) Layer 1; (**b**) Layer 2; (**c**) Layer 3.

To visualize the evolution of high-dimensional features, t-SNE is applied to reduce graph embeddings to two dimensions. The results are shown in Figure 8. From the first to the third Chebyshev convolutional layer, samples of the same leakage level become increasingly clustered, while inter-class distances grow. This confirms that deeper GNN layers progressively refine class-discriminative embeddings through neighborhood aggregation.

But as shown in Figure 8, a small number of outlier samples persist across class boundaries in each layer, indicating that some graph topologies remain difficult to fully separate. For classification, features from the third layer are preferred over shallower ones, and going beyond three layers risks overfitting or representation collapse, making the current three-layer configuration a reasonable choice. Overall, the model effectively extracts discriminative features, with clustering quality improving from shallow to deep layers. By leveraging topological associations, the GNN achieves preliminary clustering as early as the first layer, outperforming traditional methods that rely solely on intrinsic features. The t-SNE visualization clearly illustrates the feature learning process transitioning from “chaos” to “order,” providing intuitive evidence for the model’s interpretability.

**Figure 9:**
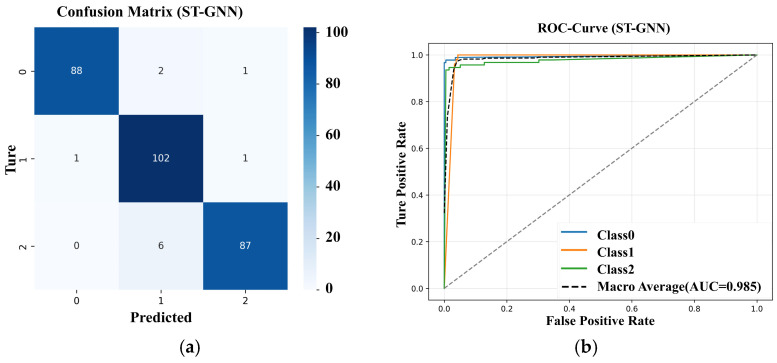
Classification results on the test set: (**a**) confusion matrix; (**b**) ROC curves.

Regarding the quantitative evaluation of classification performance, the results of the confusion matrix and ROC curve analysis shown in Figure 9 further validate the model’s superior performance. AUC (Area Under the Curve) is a key metric for measuring the area under the ROC curve, with values ranging from 0 to 1. In this experiment, the AUC values for Class 0, Class 1, Class 2, and the Macro Average all exceeded 0.98, and the ROC curves closely approached the top-left corner of the coordinate plane. This indicates that the graph neural network model can distinguish samples of different categories with very high accuracy—both at the individual leakage level and overall—demonstrating excellent reliability and discriminative power. This outstanding classification performance is attributed to the graph neural network’s effective learning of node features and its rational utilization of the graph structure, enabling the model to fully capture the subtle differences between samples of different leakage levels.

### 4.2. Statistics of Multiple Training Results

To comprehensively evaluate the performance stability and data adaptability of the graph neural network in the task of pipeline leakage level identification, multiple groups of experiments were conducted using datasets of different total sizes. Five independent datasets were constructed, with total sample sizes of 960, 840, 720, 600, and 480, respectively. The dataset of 960 samples is the complete collection described in Section 4.1, while the smaller datasets are subsets of reduced scale to simulate limited-data scenarios. The model’s performance was measured comprehensively through multi-dimensional indicators such as accuracy, F1 score, AUC, and MCC. All experiments follow the unified configuration described in Section 4.1.

As shown in Table 2, the model achieves generally satisfactory performance across most groups, yet no strictly monotonic improvement with increasing dataset size is observed. For the two groups with a dataset size of 960, the training, validation, and test accuracies fall between 0.9611 and 0.9715, with corresponding AUC values approximately ranging from 0.985 to 0.989 and MCC values from 0.94 to 0.96. These results indicate that large-scale data provides relatively stable and high-quality feature learning, though perfect classification is not attained under this configuration.

Table 2 also reveals notable fluctuations in medium-scale groups, namely those with sizes 840 and 720. For instance, Group 3 achieves a test accuracy of 0.9048 and an AUC of 0.9251 at a size of 840, while Group 4 with the same sample size drops to a test accuracy of 0.8421 and an AUC of 0.9133, suggesting that data quality and feature distribution may vary across random splits even when the sample count is identical. Among the groups with a size of 720, Group 5 yields a test accuracy of 0.8756 and an AUC of 0.9721, outperforming Group 6 which has a test accuracy of 0.8778 and an AUC of 0.9578, indicating that some splits produce more discriminative features.

For small-scale groups, as shown in Table 2 (sizes 600 and 480), performance does not always degrade monotonically. Group 8 achieves a test accuracy of 0.9009 and an AUC of 0.9399 at a size of 600, surpassing some medium-scale groups, whereas Group 7 with the same size shows lower metrics with a test accuracy of only 0.8194. This demonstrates that with appropriate feature distribution, the model can still achieve competitive results even with fewer samples.

However, Table 2 also indicates a potential risk of overfitting in several groups, such as Group 5, Group 7, and Group 9, where validation and test accuracies are noticeably lower than training accuracies. This issue, though already partially mitigated by the dropout and weight decay employed in the current training configuration, may be further alleviated through stronger regularization or data augmentation in future work. Overall, based on the analysis of Table 2, the graph neural network exhibits robust and feasible identification capability across various data scales, though performance is influenced by both dataset size and the intrinsic quality of feature distribution. This provides a practical reference for selecting training schemes according to available data resources.

### 4.3. Ablation Experiments

To verify the effectiveness of each module, this study designed network layer ablation experiments by removing different numbers of intermediate layers to gain a deep understanding of their specific impacts on overall model performance.

**Figure 10:**
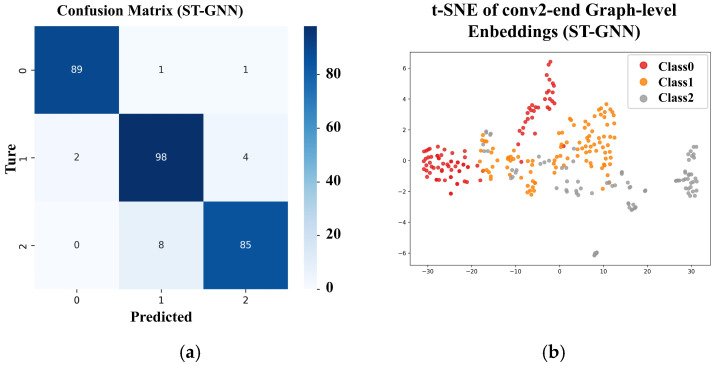
Results of the two-layer Chebyshev graph convolutional network: (**a**) confusion matrix; (**b**) t-SNE visualization of the last layer.

Figure 10 presents the results of the two-layer network, which achieves a global classification accuracy of 94.44%, a reasonably high level. The confusion matrix indicates that Class 0 is recognized almost perfectly, with only a handful of misclassifications. However, the two-layer architecture exhibits clear limitations compared to the three-layer model. Class 2 becomes the performance bottleneck: eight samples of Class 2 are misclassified as Class 1, as shown in the confusion matrix. The t-SNE visualization further reveals that while the three classes are largely separable, a notable overlap exists in the boundary region between Class 1 and Class 2, where some Class 2 samples are embedded close to the Class 1 cluster. More critically, compared to the three-layer case, the two-layer model suffers from significantly more severe embedding overlap in the inter-class boundary areas, increasing the potential for misclassification. This indicates that the reduced network depth impairs the model’s capacity to resolve subtle feature differences between similar leakage levels, particularly between Class 1 and Class 2. Consequently, although the two-layer model achieves competitive overall accuracy, it is less reliable for distinguishing ambiguous boundary samples than the three-layer counterpart.

**Figure 11:**
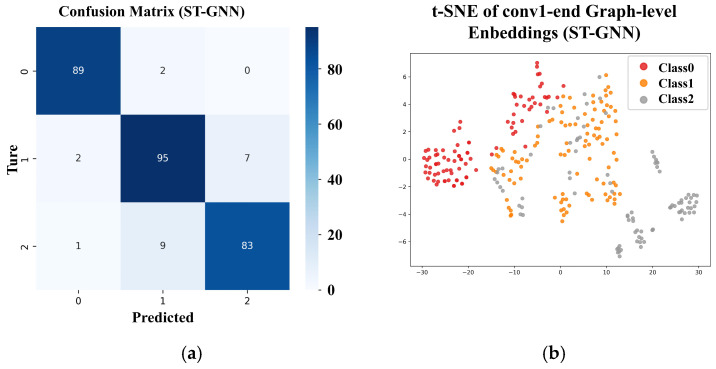
Results of the single-layer Chebyshev graph convolutional network: (**a**) confusion matrix; (**b**) t-SNE visualization of the last layer.

Figure 11 shows the results of the one-layer network, which achieves a test accuracy of 92.71% and an AUC of 0.9799. The confusion matrix reveals that Class 0 is still recognized relatively well, with 89 correct predictions out of 91 samples. However, the one-layer architecture exhibits the most severe inter-class confusion among all three models. Notably, Class 1 and Class 2 are heavily mixed: 7 samples of Class 1 are misclassified as Class 2, and 9 samples of Class 2 are misclassified as Class 1. The t-SNE visualization confirms that the embeddings of Class 1 and Class 2 overlap substantially, making their boundary nearly indistinguishable. Compared to the two-layer model, which also showed some confusion between Class 1 and Class 2 (8 misclassifications from Class 2 to Class 1), the one-layer network suffers from even greater bidirectional misclassification, indicating a complete loss of fine-grained discriminative power. In contrast, the three-layer model achieved perfect separation with no inter-class errors. These results demonstrate that reducing the network depth to a single layer severely impairs the model’s ability to extract class-specific features, especially for similar leakage levels. While the overall accuracy remains above 90%, the high degree of confusion between Class 1 and Class 2 renders the one-layer model unreliable for practical leakage level identification tasks where distinguishing adjacent risk levels is critical.

Table 3 compares the three-layer, two-layer, and single-layer Chebyshev graph convolutional networks in terms of parameter count, inference time, and classification metrics. All results follow the unified configuration described in Section 4.1. As the number of layers increases, both parameters and inference time rise moderately. The three-layer model contains 32,611 parameters and requires 2.58 ms per sample for inference, which remains lightweight and suitable for embedded deployment.

In terms of classification performance, the three-layer model achieves the best and most stable results across two independent runs, with test accuracies of 96.18% and 96.73%, F1 scores above 0.96, AUC values near 0.99, and MCC values above 0.94. In contrast, both the two-layer and single-layer models exhibit either significant instability or inferior absolute metrics, as detailed in Table 3. More importantly, the shallower networks suffer from severe confusion between adjacent leakage levels (Class 1 and Class 2), as discussed in Section 4.3.

Overall, Table 3 confirms that the three-layer model delivers superior and robust classification performance while retaining a lightweight design, making it the preferred choice for practical leakage level identification tasks.

### 4.4. Comparative Analysis

To comprehensively evaluate the classification performance of the proposed ST-GNN, we compare it against five representative baseline methods that span the commonly used paradigms in pipeline leakage detection: 1D-CNN, 2D-CNN, 2D-CNN with a channel attention mechanism (2D-CNN-AT), a temporal convolutional network (TCN), and a traditional feature-based support vector machine (F-SVM). Convolutional neural networks have been widely adopted for pipeline leakage identification by learning patterns from time-series or time-frequency representations [11,12,40], while attention mechanisms have been introduced to enhance feature discrimination [13]. TCN has recently demonstrated effectiveness in capturing long-range dependencies in fault diagnosis tasks [41], and SVM-based classifiers remain a strong baseline when combined with hand-crafted acoustic features [42].

It is important to note that pipeline leakage detection methods rely on fundamentally different physical principles and employ varied data acquisition configurations, leading to significantly different signal characteristics across scenarios [1,2,32,33,37]. Consequently, a method that performs well in one setting may not transfer directly to another. The following comparison is therefore conducted strictly on the utility-tunnel acoustic dataset described in Section 2, ensuring a fair evaluation under this specific and challenging scenario.

All models are evaluated under the identical data split (0.4 training, 0.3 validation, 0.3 test) described in Section 4.1. For the deep learning models, the number of layers or blocks is kept comparable to the three-layer architecture of ST-GNN, and key hyperparameters are individually tuned to ensure a fair comparison.

As shown in Table 4, the proposed ST-GNN achieves the highest scores across all classification metrics among the deep learning models, with a test accuracy of 96.73%, an F1 score of 0.9653, and an MCC of 0.9621. It also outperforms the traditional F-SVM in F1 and MCC, although the latter achieves a marginally higher AUC (0.9916 vs. 0.9892).

**Figure 12:**
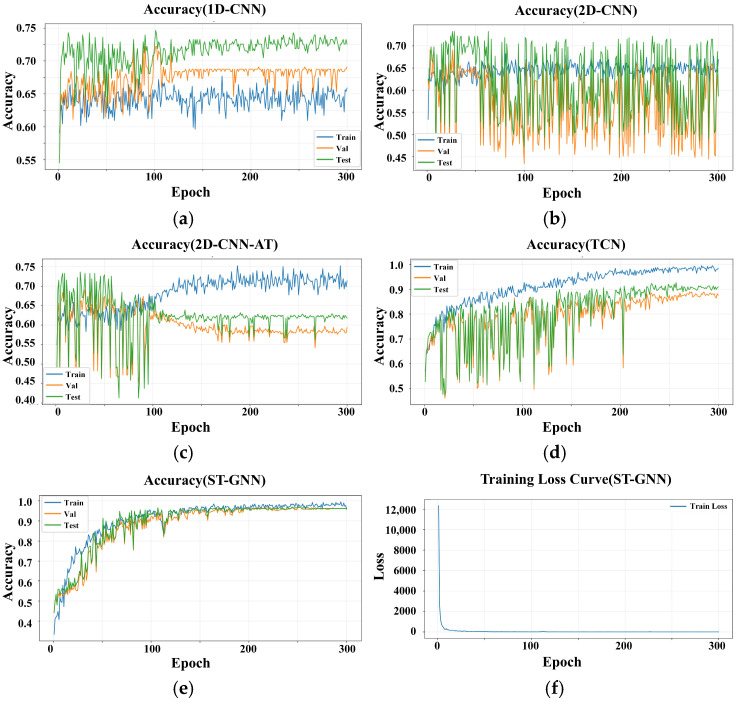
Training and validation curves of compared models: (**a**) 1D-CNN accuracy; (**b**) 2D-CNN accuracy; (**c**) 2D-CNN-AT accuracy; (**d**) TCN accuracy; (**e**) ST-GNN accuracy; (**f**) ST-GNN loss.

The unsatisfactory performance of the CNN-based and TCN baselines can be fundamentally attributed to the structural mismatch between fixed-grid convolutional architectures and the inherently non-Euclidean characteristics of leakage acoustic data. Leakage signals exhibit both local temporal dependencies and non-local spectral topological relationships—such as harmonic structures and resonance patterns—that transcend the capability of regular grid- or sequence-based convolutions. Figure 12 presents the training and validation curves of all deep learning models, providing a direct view of their convergence behavior and generalization capability.

As observed from Figure 12a–c, all three CNN-based models exhibit significant limitations. The 1D-CNN is constrained by its unidimensional architecture, with training accuracy plateauing around 73%. The 2D-CNN shows the worst convergence, with severe training oscillations and high parameter redundancy, yielding a test accuracy of only 66.60%. With the addition of channel attention, 2D-CNN-AT exhibits moderate overfitting—validation metrics fluctuate heavily and fail to generalize to the test set. These behaviors stem from the fact that CNN-based methods must reshape time-frequency data into fixed-dimensional grids, inflating parameter counts while remaining inherently incapable of capturing irregular topological correlations among time frames.

Figure 12d shows that TCN, by employing dilated convolutions to expand the receptive field, achieves the best baseline performance with a test accuracy approaching 91%. Nevertheless, its training curves still reveal mild overfitting and initial convergence oscillations, indicating that purely sequential modeling without explicit spectral similarity modeling is insufficient to fully separate adjacent leakage levels.

In contrast, as shown in Figure 12e, the proposed ST-GNN demonstrates highly stable convergence: the training, validation, and test accuracy curves rapidly converge and remain tightly aligned throughout training, with no observable overfitting. The final test accuracy stabilizes at 96%, significantly outperforming all baselines. This is achieved by explicitly modeling the intrinsic signal topology through joint construction of temporal and K-nearest neighbor edges, enabling the three-layer Chebyshev graph convolutional network with 129-dimensional node features to extract discriminative features within a lightweight architecture.

Figure 12f presents the ST-GNN training loss curve. The loss drops sharply during the early stage and smoothly approaches zero after approximately 120 epochs, with no abnormal fluctuations or rebounds throughout. This clean convergence profile confirms the stability of the optimization process and the effective convergence of model parameters.

Table 5 compares the model complexity and inference efficiency of all methods. ST-GNN contains 32,611 parameters with a per-sample inference time of 2.58 ms, striking a favorable balance between accuracy and efficiency. Although 1D-CNN has the fewest parameters (8259) among deep learning models, its test accuracy is only 72.30%, rendering it impractical for this task. 2D-CNN-AT suffers from the longest inference time (5.94 ms) due to the extra computational overhead of the attention module, yet its accuracy remains low. TCN, the strongest baseline in terms of accuracy, requires 43,843 parameters—34% more than ST-GNN—while still falling short by over 6 percentage points in test accuracy. F-SVM achieves the fastest inference (0.053 ms) with only 19 support vectors, but as a traditional method it lacks end-to-end feature learning capability and its F1 and MCC are lower than ST-GNN. Overall, ST-GNN achieves the best accuracy with moderate complexity and low latency, making it well-suited for edge deployment in underground utility tunnel monitoring systems.

## 5. Conclusions and Future Directions

This paper addressed the challenge of fine-grained identification of gas pipeline leakage level in urban underground utility tunnels by proposing a lightweight spatial–temporal graph neural network (ST-GNN). The core innovation lies in the per-sample graph construction strategy: unlike conventional approaches that treat acoustic signals as fixed-grid spectrograms or sequential time series, each signal is independently transformed into a graph where STFT time frames serve as nodes, and temporal neighborhood edges together with K-nearest neighbor edges explicitly encode both local temporal dynamics and non-local spectral similarities. This graph representation naturally captures the non-Euclidean topological structure inherent in leakage acoustics—a capability fundamentally absent in CNN- or RNN-based methods. Building upon this, a three-layer Chebyshev graph convolutional network with only 32,611 parameters and 2.58 ms inference time per sample is designed, achieving an effective accuracy-efficiency balance suitable for edge deployment.

Experimental results on a real utility tunnel simulation platform demonstrate that ST-GNN achieves a test accuracy of 96.73%, an F1 score of 0.9653, and an AUC of 0.9892 in the three-level leakage classification task. t-SNE visualization confirms progressive feature separation from complete mixing to distinct clustering, while ablation experiments validate the optimality of the three-layer architecture. Comparative experiments with 1D-CNN, 2D-CNN, 2D-CNN-AT, TCN, and F-SVM further reveal that the graph-based paradigm fundamentally overcomes the structural mismatch between fixed-grid convolutions and the non-Euclidean spectral topology of leakage signals, delivering superior convergence stability and classification accuracy. Multi-scale training statistics also verify the model’s robustness under limited data conditions.

The main contribution of this work is the introduction of per-sample spatial–temporal graph construction and Chebyshev graph convolution into gas pipeline leakage level identification in underground utility tunnels, filling a research gap in graph-based methods for this specific scenario. The proposed lightweight design reconciles high accuracy with low computational cost, facilitating subsequent deployment on edge computing platforms. Future work could be conducted in the following directions: First, extending this method to leakage detection tasks for other municipal pipelines, such as water supply pipelines, to verify its cross-domain transferability; second, refining and optimizing the algorithm and embedding it into the RK3588 device platform for operational use.

## Figures and Tables

**Table 1:** Specifications of pipeline defect sample sizes.

Leak Type	Diameter /mm	Physical Image	Leak Type	Size /mm	Physical Image
Hole	0.8	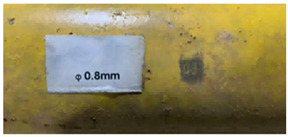	Slit	0.75 × 5	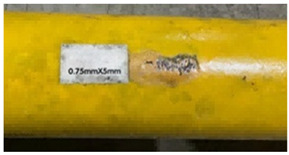
Hole	1	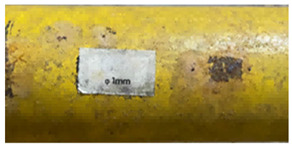	Slit	0.75 × 8	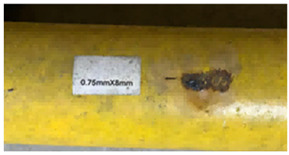
Hole	3	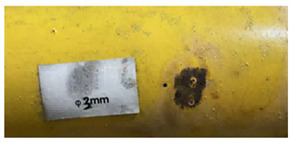	Slit	1 × 2	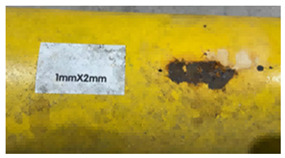
Hole	5	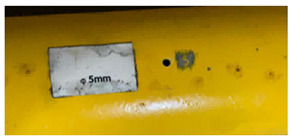	Slit	1 × 5	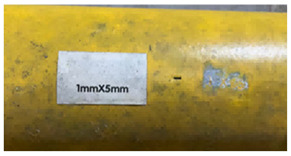
Hole	8	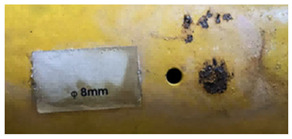	Slit	1 × 8	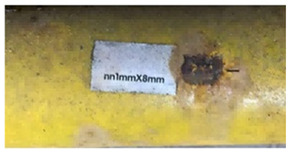
Hole	10	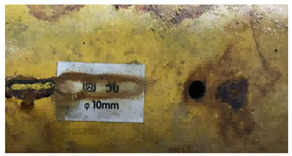	Slit	1 × 10	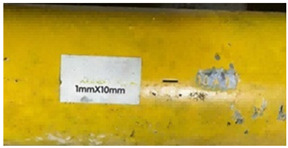

**Table 2:** Classification performance under different dataset sizes.

No.	Dataset Size	Training Accuracy	Validation Accuracy	Test Accuracy	F1 Score	AUC	MCC
1	960	0.9618	0.9645	0.9611	0.9623	0.9847	0.9430
2	960	0.9715	0.9698	0.9673	0.9653	0.9892	0.9621
3	840	0.9024	0.9015	0.9048	0.9023	0.9251	0.8537
4	840	0.8412	0.8631	0.8421	0.8456	0.9133	0.7688
5	720	0.9340	0.8744	0.8756	0.8732	0.9721	0.8359
6	720	0.8756	0.8866	0.8778	0.8769	0.9578	0.8181
7	600	0.9037	0.8425	0.8194	0.8201	0.9513	0.7906
8	600	0.9000	0.9013	0.9009	0.9002	0.9399	0.8508
9	480	0.8742	0.8542	0.8633	0.8627	0.9364	0.8071
10	480	0.8888	0.9017	0.8879	0.8918	0.9423	0.8352

**Table 3:** Ablation study on network depth: parameter count, inference time, and classification metrics.

Layers	Total Parameters	Single Sample Reasoning Time/ms	F1_Score	Test Accuracy	AUC	MCC
3	32,611	2.5766	0.9623	0.9618	0.9847	0.9430
3			0.9653	0.9673	0.9892	0.9621
2	27,971	2.2959	0.9453	0.9444	0.9758	0.9166
2			0.8271	0.8220	0.8914	0.7901
1	25,027	1.3441	0.9030	0.9026	0.9345	0.8261
1			0.9281	0.9270	0.9799	0.8905

**Table 4:** Classification performance comparison of different models.

Model Type	Training Accuracy	Validation Accuracy	Test Accuracy	F1 Score	AUC	MCC
ST-GNN	0.9715	0.9698	0.9673	0.9653	0.9892	0.9621
1D-CNN	0.7326	0.8280	0.7230	0.7336	0.8778	0.6339
2D-CNN	0.6701	0.7407	0.6660	0.6818	0.8494	0.5158
2D-CNN-AT	0.7326	0.8581	0.7214	0.7324	0.8695	0.6486
F-SVM	0.9444	0.9460	0.9464	0.9458	0.9916	0.9169
TCN	0.8993	0.9064	0.8987	0.9013	0.9842	0.8496

**Table 5:** Model complexity and inference speed comparison.

Model Type	Total Parameters	Single Sample Reasoning Time/ms
ST-GNN	32,611	2.5766
1D-CNN	8259	1.9632
2D-CNN	24,003	1.7192
2D-CNN-AT	27,687	5.9441
F-SVM	19	0.0534
TCN	43,843	2.3627

## Data Availability

The datasets presented in this article are not readily available because the data are part of an ongoing study and due to security and confidentiality restrictions of underground civil air defense projects limitation. Requests to access the datasets should be communicated to the corresponding author.

## Supplementary Materials

The following supporting information can be downloaded at: https://www.mdpi.com/article/10.3390/s26134114/s1.
